# Evidence based practice in clinical physiotherapy education: a qualitative interpretive description

**DOI:** 10.1186/1472-6920-13-52

**Published:** 2013-04-11

**Authors:** Nina R Olsen, Peter Bradley, Kirsten Lomborg, Monica W Nortvedt

**Affiliations:** 1 Faculty of Health and Social Sciences, Centre for Evidence Based Practice, Bergen University College, Bergen, Norway; 2 Director of Public Health Development, Public Health Wales, Cardiff, UK; 3 Faculty of Health Sciences, Department of Public Health, Aarhus University, Aarhus, Denmark

**Keywords:** Evidence-based practice, Evidence-based medicine, Evidence-based physiotherapy, Implementation, Clinical education, Clinical placements, Clinical instruction, Clinical supervision, Qualitative research, Focus group interviews

## Abstract

**Background:**

Health care undergraduate students are expected to practice evidence-based after they graduate. Previous research indicates that students face several problems with transferring evidence-based practice to real patient situations. Few studies have explored reasons for this. The aim of this study was to explore beliefs, experiences and attitudes related to third year students’ use of evidence-based practice in clinical physiotherapy education among students, clinical instructors and visiting teachers.

**Methods:**

In total, six focus group interviews were conducted: three with 16 students, two with nine clinical instructors and one with four visiting teachers. In addition, one individual interview and one interview in a pair were conducted with clinical instructors. Interviewing three different participant-categories ensured comparative analysis and enabled us to exploit differences in perspectives and interactions. Interpretive description guided this process.

**Results:**

Four integrative themes emerged from the analysis: “attempt to apply evidence-based practice”, “novices in clinical practice”, “prioritize practice experience over evidence-based practice” and “lack role models in evidence-based practice”. Students tried to search for research evidence and to apply this knowledge during clinical placements; a behaviour that indicated a positive attitude towards evidence-based practice. At the same time, students were novices and required basic background information more than research information. As novices they tended to lean on their clinical instructors, and were more eager to gain practical experience than practicing evidence-based; a behaviour that clinical instructors and visiting teachers often supported. Students noticed a lack of an EBP culture. Both students and clinical instructors perceived a need for role models in evidence-based practice.

**Conclusions:**

Clinical instructors are in a position to influence students during clinical education, and thus, important potential role models in evidence-based practice. Actions from academic and clinical settings are needed to improve competence in evidence-based practice among clinical instructors, and future research is needed to investigate the effect of such efforts on students’ behaviour.

## Background

Health care professionals are expected to practice evidence-based. Consequently, by the time students in health care professions graduate they need to be confident in practicing evidence-based. This is in agreement with the Sicily statement on evidence-based practice (EBP), which recommend that the necessary knowledge, skills and attitudes of EBP is incorporated into the curricula and based on the five-step model: 1) translation of uncertainty into an answerable question, 2) search for and retrieval of evidence, 3) critical appraisal of evidence for validity and clinical importance, 4) application of appraised evidence to practice and 5) evaluation of performance [1]. These EBP steps should be implemented into health care students’ clinical education; so that EBP becomes incorporated with life-long learning and patient care [1-3].

The impact of integrating EBP into clinical undergraduate education has mainly been investigated among medical students. Previous pre- and post-test studies have had some success in improving self-reported knowledge, but this has not necessarily been reflected in skills or behaviour. American medical students reported using all EBP skills more frequently and applied them regularly to clinical care after taking part in EBP seminars, then again their self-perceived skills did not match levels of performance on a skill test [4]. Similarly, when another group of American students were introduced to EBP during their primary care rotation, understanding of EBP and comfort with critical appraisal of research evidence improved, while reported use of information sources was unchanged [5].

Use of sources and literature searching skills among medical students has been the focus of several EBP evaluations, although the results vary. In a pre- and post-test study, American medical students improved both self-reported and actual searching skills after training via an on-line curriculum in EBP [6]. More recent studies have been less successful. For example, Malaysian senior medical students did not change their information-seeking practices after being exposed to a clinically-integrated EBP curriculum in a pre- and post-test study; the majority still preferred to first consult another individual for their clinical queries [7]. In a randomised controlled trial, Australian medical students did not improve their literature searching skills after a formal workshop in EBP, although they did improve confidence and ability to construct clinical questions and increased their awareness of information resources [8].

Finding research evidence has also been identified as a problem in surveys among undergraduate medical students. Malaysian medical students’ searching was inefficient, and they reported of performing searches in single journals rather than clinical databases when they had clinical questions [9]. MEDLINE searching was not prioritised among German medical students during clinical clerkship [10]. On the other hand, 67% of American medical students that participated in a survey reported that UpToDate was their primary educational resource when admitting patients or preparing for rounds [11]. Nevertheless, more than 30% of these students reported still using other primary sources such as another resident doctor, online sources and books.

Few studies have investigated the impact of integrating EBP into clinical undergraduate education outside medical education. Nursing students participating in a controlled pre- and post-test study reported of positive attitudes towards EBP, but poor competence and confidence in several EBP skills after participating in an interactive and clinically integrated teaching strategy [12]. More positive trends are evident from two pilot studies (one-group, pre- and post-test design). When use of a mobile device for critically appraising a clinical guideline in clinical settings was piloted among English nursing students, students rarely used this device; nonetheless, they improved EBP knowledge and skills, including the appraisal of clinical guidelines [13]. Chinese nursing students significantly improved their EBP knowledge, attitudes, beliefs and behaviour after participating in a pilot program during their clinical education [14]. Similar findings were found in pre- and post-test studies within rehabilitation. Entry-level physiotherapy students reported of significant changes in all EBP domains (knowledge, skills, attitude and behaviour) after participating in a theory-based EBP course and integrating these principles into clinical practice [15]. Athletic training students participating in lectures and clinically-integrated activities improved knowledge and confidence in EBP at post-test evaluation [16]. However, successful findings from these latter studies cannot be generalised due to the absence of control groups. In addition, the athletic training students reported of barriers related to time, resources, clinical instructors’ open mindedness and agreement with class information [16].

Similar barriers have been identified in other studies. A survey among occupational therapy students identified lack of time and fieldwork educators not practising EBP as important barriers [17]. In a recent survey, Swedish nursing students reported of less support for practising evidence-based in clinical education than in academic education [18]. Results from qualitative studies indicate that nursing students perceive a gap between theory and practice and sparse implementation of EBP as a barrier to desired learning during clinical education [19]; and medical students would use EBP to a greater extent if their clinical supervisors encouraged them or expected them to apply EBP [20,21]. To date, use of EBP among undergraduate physiotherapy students in clinical practice has not been explored.

It seems that students struggle with applying the principles of EBP in clinical settings, and “best practice” has yet to be established when it comes to integrating EBP to clinical undergraduate education, and in particular outside medical education. To achieve a better contextual understanding of necessary actions to ensure use of EBP in clinical education we decided to explore perspectives regarding students’ use of EBP among all those involved in clinical physiotherapy education. The aim of this study was to explore beliefs, experiences and attitudes related to students’ use of EBP in clinical physiotherapy education among students, clinical instructors (CIs) and visiting teachers.

### Context

This study was set up in the context of physiotherapy undergraduate education at Bergen University College in Norway. In Norway, four university colleges offer three-year bachelor programs (180 ECTS-credits) in physiotherapy [22,23]. An additional year with supervised internship in hospital practice and primary health care is required to become an authorised physiotherapist. During the three-year bachelor program students spend 30 weeks (45 ECTS-credits) in clinical placements, each placement lasting from a few weeks up to 11 weeks [22,23]. Experienced clinical physiotherapists supervise students in various clinical settings: primary health care, outpatient clinics, rehabilitation clinics, local hospitals and university hospitals [22]. CIs are responsible for students during clinical placements. Visiting teachers from the university college (academic staff) visit students at the placement once or twice, and contact students frequently via mail or telephone.

After graduation physiotherapy students should be qualified to keep up to date and use research in a critical and analytic way [23]. Thus, research methods and statistics have been essential components of the bachelor programme for years. Still, teachers at Bergen University College experienced that students in general struggled with finding and using relevant and valid information such as research evidence. As a consequence, teachers started teaching EBP in 2004, and by 2006 EBP was implemented across the three-year program. Thus, students participating in this study were, in year one, provided with a three-hour introduction to the concept of EBP, and they were encouraged to apply EBP steps in a patient report related to a real clinical scenario identified during their clinical placement. In addition, writing and searching courses and tutorials were provided. In year two, students were provided with more advanced teaching related to EBP, including critical appraisal of research articles and clinical guidelines and lectures on searching for research evidence. In total, students received 19 hours with EBP teaching this year. In year three, applying the principles of EBP was obligatory in several activities, for example when discussing problem-based clinical scenarios and when writing patient reports related to real clinical scenarios from clinical placements. In these patient reports students had to describe a patient situation and their clinical decisions. As arguments for their decisions they had to use literature, but not necessarily research evidence. Except for the patient reports in year one and year three, using EBP during clinical placement periods was not required or assessed among these students. Thus, it was uncertain whether and how students applied EBP, and whether they were positive and confident with using EBP in real patient situations.

## Methods

### Design

Interpretive description strategy guided the process of capturing patterns and themes within subjective perceptions and experiences related to students’ use of EBP during clinical education. Interpretive description is an inductive approach inspired by grounded theory, naturalistic enquiry, ethnography and phenomenology ([24], p. 6). By use of constant comparative analysis interpretive description offers a coherent strategy to conceive, design and implement research capable of generating new insight into clinical settings ([25], p.17). To ensure comparative analysis and the possibility of exploiting differences in perspectives and interaction, we invited the different participant-categories involved in clinical education to take part in this study. Conducting interviews with different participant-categories is described as a multi-category design ([26], p. 31); a strategy that allows comparison within and between participant-categories.

Interpretive description is a suitable research strategy to study phenomena in practical disciplines such as nursing, teaching and management [24,27,28]. In addition to developing interpretive descriptions of people’s experiences, this approach aims to produce knowledge and a contextual understanding that can be put to direct applied use in clinical practice ([25], p. 33, 36).

### Participants

A purposive sample consisting of three different types of participant-categories; third year physiotherapy students, physiotherapists functioning as CIs and academic staff functioning as visiting teacher, were invited via e-mail during January – April 2008. The Department of Physiotherapy at Bergen University College provided a complete list of all study participants.

In total, 32 persons between the ages of 21 and 55 years participated in the study (16 students, 12 CIs and four visiting teachers) (Table 1). Only five of the participants were men. Most students were interviewed during their final 10-week clinical placement during spring 2008. CIs were first and foremost recruited from hospitals located geographically close to Bergen, due to time and costs of travelling. Hospitals outside Bergen are situated far apart, and often there were only one or two CIs situated at these hospitals. Thus, for practical reasons CIs from hospitals outside Bergen were invited to take part in individual interviews.

**Table 1:** Characteristics of the participants

**Characteristics**		**Focus groups: Students**	**Focus groups: Clinical instructors**	**Focus group: Visiting teachers**	**Individual interviews: Clinical instructors**
Invited/participated		55/16	21/9	7/4	14/3
Number of interviews		3	2	1	2
Geographical location					
	Close to Bergen	12	9	4	
	Outside Bergen	4			3
Time of interview					
	During placement	12			
	After placement	4	9	4	3
Sex					
	Men	1	2	2	
	Women	15	7	2	3
Age					
	20-29	15	1		
	30-39	1	4		2
	40-49		3	2	1
	< 50		1	2	
Clinical experience (yrs)					
	0-4		1		1
	5-9		4		1
	10-19		3		
	< 20		1		1
Education					
	Master		1	2	1
	PhD or equivalent			2	
Courses					
	Method (9–15 ECTS)		2	4	
	EBP (15 ECTS)		1	2	

### Data collection

We conducted six focus group interviews with physiotherapy students, CIs and visiting teachers. Focus group interview was the primary data collection strategy in this study, although, we also conducted one individual interview with one CI and one interview with a pair of CIs from hospitals outside Bergen. These latter interviews were conducted in order to gain insight also into the perspectives of CIs situated at hospitals outside Bergen that were not university hospitals or geographically close to Bergen University College or academic centres of excellence.

All interviews were conducted during spring 2008 (Table 1). Participant-categories were not mixed due to a possible expertise and power differential, which is known to make some participants reluctant to talk ([26], p. 27). In each focus group interview, study participants were homogenous with regard to background as student, CI or visiting teacher. Participants at clinical placements in Bergen were not mixed with participants at clinical placements outside Bergen (students and CIs).

In this study, we anticipated that some of the study participants would have limited knowledge and experience with the topic EBP. We believed that encouraging interaction through focus group interviews could inspire the participants to consider and talk about EBP and elaborate on this topic. Thus, by encouraging interaction in groups we aimed to facilitate the collection of rich and meaningful data. To facilitate interaction we made an effort to create a comfortable atmosphere and an informal environment where study participants could feel comfortable talking freely about the topic EBP. Efforts were also made to make participation as unproblematic as possible for the study participants. Therefore, all interviews were scheduled at lunchtime and held at convenient locations such as meeting rooms at clinical placements or on the University College campus.

For all interviews we used a semi-structured interview guide that was based on the aim of this study, our current contextual understanding of the problem and previous relevant research. The interview guide consisted of a standardised set of questions with five key topics: 1) perceptions of EBP, 2) students’ information need during clinical education, 3) students’ use of research evidence during clinical education, 4) facilitators and barriers to EBP during clinical education and 5) how to best promote EBP in clinical education in the future. In addition, we identified a list of probes and issues that we believed were essential to discuss, although these issues were only brought up if they did not occur in the natural discussion. Prior to the interview sessions, all study participants were asked to fill in a form asking for general demographic data. All interview sessions lasted 1–2 hours and were digitally recorded. The interviews were transcribed verbatim by a secretary/student, who received clear instructions about the procedures and purposes of transcriptions.

All focus group interviews were moderated by NRO and co-facilitated by two different assistant moderators. There was a potential disadvantage that the conversations were dominated by more experienced members of the groups, or that some did not feel that this was a situation where they could speak freely. Therefore, summaries of main issues that were brought up during the discussions were e-mailed to the participants of the focus groups for comments (member check).

All interviewers were familiar with the topics in the interview guide as they worked with integrating EBP either in academic or clinical settings. In addition, they were all experienced facilitators, had previous experience as physiotherapists and experience from teaching physiotherapy. All researchers involved in the project were positive towards EBP, and some were also experienced in teaching EBP (NRO and PB). A researcher team, with different nationality, background and expertise from both education and clinical practice, ensured investigator triangulation throughout the project.

### Ethical considerations

All the involved institutions; Bergen University College; Haraldsplass Diakonale Hospital; and Haukeland University Hospital, supported the project. The Regional Committee for Medical and Health Research Ethics, Western-Norway, exempted this study from review, because the study did not include medical or biomedical aspects. The Norwegian Social Science Data Services (NSD) approved the study. In keeping with the approval from NSD, we obtained written informed consent prior to all interviews, stored recordings appropriately to preserve confidentiality and deleted recordings after end of project. Anonymity of participants was preserved by eliminating names from transcripts, and since only three of the participants were men all participants were referred to as “she” in this article.

### Analysis

A combination of NVivo and Word processing was used to aid the constant comparative analysis of the focus group interviews throughout a series of technical and intellectual operations: 1) immersion in the transcripts, 2) development of an initial template, 3) organisation of the data based on the template, 4) condensing and reflecting, 5) comparing and contrasting within interviews with similar participant-categories and finally 6) comparing and contrasting between interviews with different participant-categories. Following these phases made it possible to gain a comprehensive insight to our data, and also helped us to consider similarities and differences with respect to a wide range of dimensions among the different interviews. By comparing and contrasting both within and between interviews we were able to generate patterns and themes within the data set overall.

Immersion in the data was achieved during short debriefing sessions after each interview, listening to all the recordings between each interview and reading the summaries from the discussions. Based on these activities we evaluated whether it was necessary to modify the interview guide for the next interview. In this way, the process of data analysis commenced as soon as data collection began, and our early analytical assumptions were pursued in the on-going data collection. Moreover, immersion in the data was also achieved through reading all transcripts repeatedly and writing marginal remarks on the transcripts, either to query segments of data or to point to important issues, or potential themes or patterns. In this way, we developed a sense of the whole, as described by Thorne ([25], p.143).

According to Thorne ([25], p. 144) some coding is needed in order to sort and organize information into a manageable form. We followed the template approach as developed by King [29] and described by Crabtree and Miller [30]. This approach involves constructing a hierarchical structured list of codes in the form of a template, representing themes identified in the transcripts; higher-level codes representing potential broad-based themes and lower-level codes representing more narrowly focused themes [29]. Furthermore, main questions from the interview guide can be used as a starting point for constructing higher-level codes, and additional questions and probes as lower-level codes. Defining a number of codes or themes relevant to the aim of the project a priori is characteristic of template analysis [31]. However, these themes are tentative, and researchers are open to modifying or deleting them as the template is developed, as opposed to coding categories used in quantitative content analysis [31]. Thus, through reading and analysing the transcripts, a code could be added, because an issue in the transcripts relevant to the aim of the study is not covered by a code, or a code could be deleted because researchers do not see the need to use it [29]. Codes could also be re-defined at a higher or lower level [29]. Codes that cut across the hierarchical organization are so called “integrative themes” ([31], p. 334).

Development of our template was based on topics from the interview guide, in addition to other potential codes that emerged during the immersion in the data. The template was used for the initial organising of the text from the transcripts, carried out by NRO. NRO, PB and KL carried out the other phases of the analysis, where the template was continuously revised until most issues in the transcripts, relevant to the aim of the study, were covered by a code. In the final template, five codes were defined: 1) understanding/knowledge of EBP, 2) students’ behaviour, 3) CIs’ behaviour, 4) barriers and facilitators 5) future interventions. These were subdivided into further codes. In addition, we were able to identify several integrative themes that cut across this hierarchal template, due to the process of first condensing, or summarising the data, and further reflecting on the organisation: did the text really represent the codes in the template, did alternative interpretations emerge and were any issues missing from the discussions. Furthermore, comparing and contrasting the coding within and between the interviews with the different participant-categories enabled a more holistic analysis where we identified the final patterns and themes within the overall data set.

In the final phase of inquiry, the individual interview and the interview in pairs served as an external clarification and validation for the emerging conclusions from the focus group interviews.

## Results

**Figure 1:**
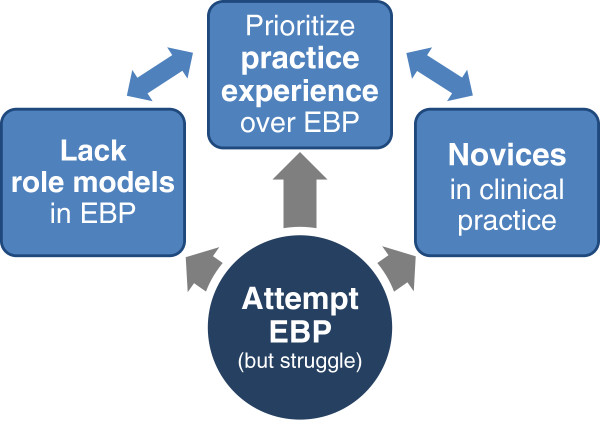
Conceptual model of students attempting EBP.

The focus of the interviews was beliefs, experiences and attitudes related to students’ use of EBP during clinical placements. A range of themes relating to this topic were generated in the analysis. It is not possible to deal with all these themes in equal depth, and therefore, we have chosen to concentrate here on findings related to the identified integrative themes: “attempt to apply EBP”, “novices in clinical practice”, “prioritize practice experience over EBP” and “lack role-models in EBP”. These themes address the aim of the article in a more holistic way, as they were identified across all codes in all interviews. Figure 1 illustrates the relationship between the integrative themes.

Participants in this study experienced that students attempted to apply EBP by searching and using research evidence in clinical settings, but struggled for various reasons. As students were novices in physiotherapy they experienced that learning new routines at the clinical placement took time, and they found it more convenient to use CIs and other persons as their information sources, compared to finding research evidence on their own. Students also prioritized gaining practice experience over EBP and seemed to consider EBP as a non-clinical activity. Several CIs and visiting teachers had similar attitudes. In general, students noticed ambivalent attitudes towards EBP and little EBP behaviour at the clinical placements, indicating a lack of role-models in EBP. Students discussed the importance of an evidence-based culture, and participants saw the need for role models in EBP.

### Attempt to apply EBP

Across all interviews searching for research evidence and using research evidence were the most common student EBP activities that were mentioned. In interviews with students and CIs, searching for research evidence was mentioned as a core EBP activity among students during clinical placements.

#### Searching for research evidence

CIs believed that students were fairly confident at searching for research evidence. Nearly all CIs specifically mentioned that students were good at searching for information on the Internet, and students were also good at asking questions. One CI expressed the following:

“My impression is that they [students] are much better at searching than I am, and probably ever will be [laughter]. But it is amazing how many tools that are available. So, I had nothing to contribute with when it came to searching.” (CI 2, Interview 5)

Some students also mentioned that they helped their CIs navigating on the Internet, and some students shared articles with their CIs. Still, it seemed that students needed to further improve their searching skills. Only a minority of the searching situations described in the interviews were related to specific clinical problems, as for example “What is the effect of exercise among patients suffering from heart failure?” or “What is the prognosis among patients with nerve damage?” Instead, several examples from interviews with both students and CIs, demonstrated that students’ information need was often less specific without specifying whom the patient was, or what kind of diagnosis or therapy that was of interest. One CI described a situation where a student tried to search for “more knowledge” about phantom limb pain:

“I have one example related to my student. It is not a completely unfamiliar problem, but in relation to gathering more knowledge about phantom limb pain, I shared some information with my student from books and articles. In addition, the student had also done some searching for more articles, more knowledge.” (CI 4, Interview 4)

Students themselves were less confident regarding their searching skills, except for one student who expressed that it was easy to use the Cochrane Library. Most students were frustrated that searching took too long time and that it was difficult. One student expressed her frustrations as follows:

“I had one patient with balance problems, and tried to find out about strength. I read a little in books about what it is [balance], and the importance of strength in under-extremities for patients with balance problems. I found some information in books, but I struggled to find research articles on this topic. I spent so long time looking, that in the end I gave up.” (Student 1, interview 1)

They also lacked the skills for finding the correct search terms and for conducting sufficiently narrow searches, as one student described:

“A while ago I had a patient with a complex pain situation in the shoulder, neck and upper trunk region. I had many findings from the clinical examination, but it was such a complex situation, and I did not know. I did not formulate a specific question, so I searched a little in relation to neck and shoulder pain, both in relation to tendinitis in shoulder and in relation to muscles in neck and shoulder. But, it became such a wide search. I tried different search terms without finding anything, and I felt that I did not have a specific plan.” (Student 2, interview 2)

Students who struggled with searching often chose alternative strategies for finding information. They used information sources such as books, medical dictionaries, Google, other web sites, local guidelines and advice (most often not evidence-based), or they asked CIs or peer students.

#### Using research evidence

Participants described various situations where students used research evidence: teaching sessions, written patient reports, discussion groups and treatment planning. Use of research evidence for the patient report, was mentioned across all interviews. This written patient report was an assignment that was due straight after the clinical placement, and should be based on students’ experiences with real patient situations from the clinical placement. Both students and CIs referred to involvement in discussion groups where use of research evidence was evident. CIs provided students with articles on given topics, such as balance in stroke patients, which they discussed together in groups. A less formal discussion between a CI and a student was triggered by a CI who gave the student four articles about how to treat lumbopelvic pain:

“[…] she [ her CI ] gave me four articles as an argument and background for treatment she was conducting. However, they were not critically appraised. Searching the Internet might reveal other conflicting articles. […] So I gave her two opposing articles, and then we got a discussion going […] about not using CPAP [continuous positive airway pressure]” (Student 4, Interview 3)

In this situation, the student and the CI attempted to apply EBP together by critically appraising different research articles relevant for clinical practice.

Some participants described students using research evidence in direct relation to treatment planning. One CI described a specific clinical situation, where she and her student discussed use of transcutaneous electrical nerve stimulation (TENS) and treatment dosage. The student presented treatment recommended in research evidence, and the CI let her student treat the patient in line with these recommendations, since she did not have any other suggestions herself. In interviews with students there were more examples of use of research evidence in real clinical situations. For example, one student reported that she used information from articles to plan an exercise sessions for patients who had suffered from a cardiac infarct. This student integrated knowledge from several randomized controlled trials with knowledge from lectures, practice experience from her CI and patient values. Another student explicitly expressed how she applied findings from an article about exercises for patients with knee arthritis:

“I selected the exercises that were relevant, since not all exercises were as relevant.” (Student 3, Interview 1)

These latter examples indicate that students apply research evidence, although they might apply findings from articles without being critical to validity or reliability. Only one student mentioned that she critically appraised research evidence during clinical placement. She critically appraised two review articles in collaboration with her CI who wanted to learn how to critically appraise. Moreover, one visiting teacher believed that students lacked sufficient skills in research method to fully understand how to critically appraise articles. A lack of critical appraisal skills was also reflected in the student interviews. A couple of students expressed their frustrations with articles covering similar topics, but concluding differently. They were also unsure about how to apply findings from articles when the real patients they met had co morbidities that were not covered in the articles.

### Novices in clinical practice

Students could be struggling with EBP, because their focus is on finding their place in clinical placements and mostly have information needs that can be answered by information from other sources than research evidence. Students being novices in physiotherapy, and needing basic information from books and other similar sources, is reflected across all interviews. For example, participants from all interviews mentioned that students used books to learn more about different diagnosis. Beginners often feel the need for “recipes”, and so did these students. One student complained that she could not find descriptions of exercises for patients who had undergone knee replacement surgery:

“I tried to look for articles related to training for patients who had undergone knee replacement surgery. I could not find any articles, which described examples of exercises […]” (Student 3, Interview 3)

In addition, descriptions across all interviews indicated that CIs also were very important sources of information for students during clinical placements; as emphasized in the following discussion between two students who described how they used to imitate their CIs:

“I felt that I leaned myself very much on what my CI said, and also what was written in the local guidelines and advice. So, when I read something and then heard she [her CI] say something similar then I did that. I was perhaps a bit bad at checking in other sources, if that was the correct way to do it.” (Student 4, Interview 3)

“It often goes that way that you see what your CI does, and you almost become a copy of that.” (Student 2, Interview 3)

Most students described situations where they asked their CIs for advice, and asking their CIs was perceived as more relevant and time efficient than searching for research evidence in databases, or even using local guidelines and advice:

“Yes, this is about the time issue. First, you sit down and search, and then you have to find something relevant, and then you have to read through the article and find out if it is good enough and applicable by using different criteria. So, it is a very long process, compared to using the local guidelines and advice or to asking your clinical instructor.” (Student 1, Interview 1)

### Prioritize practice experience over evidence-based practice

Another reason for students struggling to apply EBP could be their focus on gaining practical experience while at clinical placements; reflected across all interviews. Both CIs and visiting teachers described busy clinical placements where students had too many other things to do than for example searching for research evidence. One visiting teacher believed that clinical placements are about gaining practical experience, and expressed that:

“[…] students need practical tools; they do not care about searching for articles, this is not their main concern.” (Visiting teacher 4, Interview 6)

This is also reflected in the interviews with students and CIs. One CI talked about a situation where she encouraged her students to spend time searching, using her computer; instead, both students preferred to spend time with their CIs and patients. Students experienced that to get into routines and activities at the hospital took time and handling patients seemed to be more than enough for students. Therefore, most visiting teachers and CIs believed that students had neither time nor energy to focus on EBP. They believed that too much is expected from the students. In the words of one CI:

“I think that we are asking a lot. Think of us; we are in a safe situation and have been working for a while. Even we are pretty worn out at the end of the day, and I do see that they [students] are exhausted before the end of the day […]. After all they have a private life as well. And if they are dead tired when they finish for the day and then go back home to start searching as well, then the study program is 200%. That is tough on them.” (CI 3, Interview 5)

Several CIs did not expect students to prioritize EBP activities after working hours [08:00–15:00]. Similarly, nearly all students expressed that they did not want to spend time working evidence-based after having finished for the day. One student believed that:

“[…] it is boring to do this [searching] during working hours, since you are at clinical placements to see as many patients as possible and to be in the field, and not to sit in an office searching, something I could do at home instead, or during a study day. If I was to sit and read and then I get an offer to come along if I like, so that I can see as many patients as possible; that is why I am here, to learn. I cannot be at two places at the same time. […] I learn so much more from being with patients compared to sitting down reading […].” (Student 6, Interview 1)

One student specifically said she probably could have spent time on EBP activities during working hours, and wished for at least one study-day a week to do so. Many of the students wished for a study-day like this, but still wanted to spend most time with patients. The following citation reflects student’s ambivalence:

“[…] we need a day off during clinical practice to reflect and search. However, I also have some conflicting feelings about that, because the clinical placement is so short anyway. “ (Student 3, Interview 3)

### Lack role models in EBP

In both student and CI interviews, there were some examples of CIs who searched for or used research evidence. Some students reported of CIs who shared research evidence, for example in connection with discussion groups where CIs and students met regularly to focus on specific clinical topics (e.g. balance and postural control) and to discuss relevant articles. One CI talked about how she encouraged her student to search, and another CI described a situation where she collaborated with her student about searching for research evidence:

“I have spent some time searching for articles myself. I discussed this with the student and we printed out some articles. And all of a sudden, she remembered that she [the student] had seen other articles in other databases, and found these articles rapidly for me. So, we established a good teamwork, at a different level than the day-to-day practice. What can I say? Teamwork. In a way the student could contribute very much. There you go!” (CI 3, Interview 4)

Other persons at the clinical placement also seemed to have an interest for research evidence. In one of the student interviews, two students referred to an in-service training session at the hospital where they talked about electrotherapy and were confronted with questions about the articles. Nevertheless, students across all interviews noticed that EBP was not a routine practice at the clinical placements. Two students specifically discussed local guidelines and advice at clinical placements that were not updated and the consequences of this:

“As far as I know they do not update them [local guidelines and advice], like they regularly do every second year at another student’s clinical placement [student who is not part of this specific discussion]. It is more like they say that they will consider updating them [local guidelines and advice], but they lack time to do so […].”(Student 2, Interview 2)

So, it is not so relevant for us to go and look for articles [… ].” (Student 3, Interview 2)

“I did not feel that there was an environment, or a reason, for us to do it or take an initiative to search for research evidence. No, what might hinders us is the atmosphere for it, or the environment at work for EBP in a way [… ].” (Student 2, Interview 2)

According to several students’ descriptions, CIs’ interest and competence in EBP could be an important factor to drive change when it comes to implementing EBP in future clinical education. One student believed that an evidence-based culture at the clinical placement could influence students to see the value of EBP. For example, students’ accounts indicated that CIs ought to be good role models and set good examples, if students were to be bothered about EBP at all. In the words of one student:

“I believe that it is very important that the CIs themselves are up to date […] if students meet a CI who is not up to date then we cannot be bothered. It does not help. […] it does not help us.” (Student 5, Interview 1)

The need for improving competence in EBP among CIs was also reflected in the interviews with CIs. One CI explicitly expressed that it was necessary to focus on them in future implementation of EBP:

“I believe that you need to start with us [CIs]. And possibly influence us. If you want us to change practice while students are at clinical placements […]. So, if you want students to work evidence-based on their own, then I have to change my way of supervising or my way of working, for example.” (CI 1, Interview 4)

## Discussion

We found that students in clinical placements tried to search for research evidence and to apply this knowledge, indicating that they valued and recognised EBP as a central element of clinical practice. At the same time, our results demonstrated that students struggled to apply EBP. As novices in clinical practice, students leaned themselves to their CIs as their main source of information. In addition, they prioritized gaining practice experience over applying EBP, considering these activities as separate, rather than directly complementary. Many CIs and visiting teachers seemed to support this prioritizing. In addition, students experienced a lack of role models in EBP, and participants from all interviews recognized the need for an evidence-based culture if students were to successfully apply EBP in future clinical education.

Students’ efforts to apply EBP by searching for and using research evidence suggested that they were positive towards EBP and believed that they had some competence in EBP. These findings are consistent with results from several previous surveys among undergraduate students [17,18,32-35]. Still, students in this study seemed to struggle with finding research evidence; a problem that has been identified also in other studies [6-11]. One possible reason for students’ struggle could be that undergraduate students are at their beginning of their careers, and have less knowledge and clinical reasoning skills then their future colleagues [36]. They are beginners who need answers to basic questions; at this point of their learning curve they need “recipes”, or standard routines for how to treat patients [36], and have not reached expert performance levels in EBP [37]. Simultaneously, third year students who are very soon graduating should be prepared to practice evidence-based. Thus, it is somewhat surprising that students in this study often referred to information sources such as books and local clinical guidelines and advise (not always evidence-based) to answer their clinical questions, for example about diagnosis or descriptions of treatment regimes.

Students in this study also experienced problems with applying research evidence to real patient situations, in particularly when patients they treated had co-morbidities. Difficulties with determining how to best transfer research knowledge into treatment of the unique and individual patient, is however, also an issue that has been reported in previous qualitative studies among graduated clinicians [38,39]. Thus, it is perhaps less surprising that undergraduate students in this study found it difficult to determine if research evidence was valid and applicable; bearing in mind their modest amounts of clinical experience.

To successfully work evidence-based it is necessary to develop clinical expertise, which requires use of clinical skills and past experience [40]. Students are novices in clinical practice, whereas their CIs have developed clinical expertise. Accordingly, students perceived CIs as their primary information source during clinical placement period. One student stated that it was more convenient and efficient to turn to someone experienced than searching for evidence on her own, and others described how they imitated their CIs. Such findings do not necessarily only pertain to students, as similar findings have been described elsewhere. A study among physiotherapists and occupational therapists revealed that information from peers was considered faster and more “to the point” than other sources such as research literature that was perceived as a less important source [41]. In addition, an overview of the state of the art of research utilization in nursing and allied health suggests that interpersonal and interactive sources of knowledge, such as dialogue with colleagues, are preferred ([42], p. 261–262).

Competing demands; learning new routines and gaining practical skills often seemed to be more than enough to cope with for students during clinical placements. Our findings revealed that almost all participants believed that students should prioritize practice experience over EBP, as this was the main concern of the clinical education. Both students and CIs used lack of time as an argument for prioritizing practice experience over EBP. For example, students experienced searching for research evidence as frustrating, difficult and time-consuming, and CIs believed that students had nor the time or the energy to focus on EBP. The time issue is expected, since students consider EBP as difficult and difficult tasks obviously takes longer time. Perhaps, that is why time is considered the most common barrier among a wide range of health care professionals [14,38,39,43-54]. On the other hand, when it comes to time as a barrier towards EBP the core issue may be “the kind of time, rather than the amount of time” ([42], p. 259). Lack of time as a barrier is probably about more than lack of actual clock time, and it is a more complex issue than depicted in the literature [55]. Possibly, it is about the mental time and energy that is needed in today’s complex and busy clinical world that students meet at their clinical placements. This could explain why students felt overwhelmed, and thereby, less open for implementing the principles and steps of EBP into their clinical decision making.

Lack of sufficient EBP skills is maybe a more plausible explanation for why students prioritized practice experience over EBP and experienced difficulties. Descriptions from the interviews indicated that students applied research evidence without consideration of validity and experienced frustrations and low confidence in relation to searching for research evidence. In addition, there were few examples of students using knowledge sources such as systematic reviews or clinical guidelines; sources that are considered helpful to better seek evidence-based information [56]. Furthermore, it did not seem to help students that an increasing amount of systematic reviews and guidelines with relevance to physiotherapy are produced [57]. Our findings agree with several surveys among physiotherapists and other allied health professions, where lack of skills is cited as a common barrier to EBP [48,49,52-54,58]. Higher levels of confidence in critical appraisal or in implementing EBP have been reported in some studies [33,50,51]; however, these results might be explained by low response rates that again might reflect a lack of ability or interest in EBP among non-respondents. Previous qualitative studies among physiotherapists and occupational therapists refer to similar findings as in our study: lack of skills in critical appraisal inhibited the development of EBP [59]; lack of access, skill or time was perceived as barriers to performing computerized searches [41]; and, the scientific language was hard to understand [39]. Considering this, continuing to improve skills in searching and critical appraisal, in addition to increasing awareness of knowledge sources, might be important to promote EBP among students in clinical placements.

At the same time, students struggling to attempt EBP could be explained by the fact that the provided teaching in EBP had not been sufficiently context specific. Teaching of EBP for these students had mainly occurred in an academic setting, although some written assignments related to real clinical patient scenarios required students to apply research evidence. Accordingly, they had received little if any practical guidance in clinical settings on how to apply research evidence to real patient management. When knowledge generated in teaching is not related to the reality of practice experience this could cause a gap between practice and research [60]. A theory-practice gap could occur if course content is delivered only as separate subject domains or textbook cases are communicated as simplistic representations of complex “real-world” phenomena. More effective learning of EBP could be achieved when students are actively engaged in clinically relevant situations, such as during fieldwork and in clinical settings, as described in a qualitative study among masters of occupational therapy students [61]. Various types of evidence support clinically integrated teaching: research evidence [2,6,15,62]; socio-cultural learning theories [63] and models of teaching EBP [40]. This evidence need to be considered when planning and developing teaching interventions that aims to facilitate evidence-based patient management in future clinical physiotherapy education.

More importantly, clinically integrated EBP teaching, perhaps via CIs, could be important when considering the effect CIs can have on students. CIs are responsible for students in clinical practice and probably they are students’ closest “colleague” during clinical placements. Our findings support this as all participants described how students used CIs as their main information source. CIs naturally have a unique position serving as instructors and role models to physiotherapy students [64,65], and are most likely potential role models also when it comes to promoting EBP among students [65]. Role modeling EBP can involve asking questions aloud, finding and appraising relevant evidence, discussing aloud how the evidence will be used to plan a treatment strategy ([40], p.200). In this way learners are given the opportunity to observe how evidence can be integrated into real patient situations, without being treated as something separate. The importance of role modeling has been emphasized by findings from several qualitative studies: Norwegian medical students applied research evidence when their clinical placements tutors encouraged them to do so [20]; masters of occupational therapy students identified the relationship with their CIs to be important to their clinical learning, including the application of EBP [61]; and, Australian medical students described clinical supervisors using research evidence in real time practice as an important facilitator for EBP [21].

In this study, there were some examples of CIs role modeling EBP; indicated by descriptions of CIs who shared research evidence with their students. However, in general, participants’ descriptions indicated a lack of EBP role models and an EBP culture. This is consistent with a previous study, which found only elements of EBP when analyzing the interaction between clinical educators and physiotherapy students during supervision discussion sessions ([66], p. 3–4). The main focus of supervisory interaction tended to be practical skill [67]. Similarly, Finnish and Swedish students observed that their preceptors (CIs) were negative towards research, and they were not interested in focusing on searching for articles during clinical placements [19].

Acquiring clinical skills is of course an essential part of physiotherapy education. Then again, emphasising only this type of supervision might not facilitate critical thinking and reflective practice [67]. Clinical reasoning, research and theory are other elements that need to be integrated into clinical practice in order to move the profession “from a technical, hands-on perspective to a more academic perspective in the educational programme” ([64], p. 20). To achieve this, students need good EBP role models; a need identified by both students and CIs in this study. Students specifically mentioned that CIs who are competent in EBP could facilitate change among students, and CIs themselves believed that it was necessary to focus on them to integrate teaching of EBP in clinical education. Thus, it is seems essential that the clinical and the academic environment need to collaborate in order to improve EBP competence among CIs.

### Limitations

This interpretive description of beliefs, experiences and attitudes related to students’ use of EBP during clinical education is based on the perspective of participants from Norway, from one of four bachelor programs in physiotherapy. Studying participants from other similar institutions in Norway would probably provide more details. Furthermore, CIs situated in Bergen might differ from CIs situated outside Bergen. CIs in Bergen might have been more experienced in EBP and keener to promote EBP, given their proximity to Haukeland University Hospital and to Bergen University College, where policy statements support an evidence-based practice [68,69]. Interviewing CIs situated at hospitals outside Bergen ensured the perspective from more rural areas and perhaps less exposed to EBP. In addition, we cannot exclude the possibility that viewpoints of students, CIs and visiting teachers might vary in other international educational programs. However, we believe that our result may be transferable to other countries and different health care professions, since the results in this study are consistent with other findings.

There are pros and cons in the choice of focus groups compared to individual interviews. Focusing mainly on different perspectives and interactions in groups enabled us to “go beyond “averaging” what individual contributions might suggest”, as described by Thorne ([25], p. 132). A focused group discussion can potentially create a synergy that is not possible in individual interviews: “…a group possesses the capacity to become more than the sum of its parts…” ([26], p. 24). In this study, we experienced this synergy especially when study participants discussed their experiences and explained themselves to each other. On the other hand, focus groups are not necessarily the best situation for individuals to speak freely and it can be difficult to capture various in-depth experiences [25]. Thus, we did risk not eliciting data representing non-dominant perspectives from the focus group discussions, and there was a risk that participants felt uncomfortable expressing “politically incorrect” perspectives related to EBP. To avoid this feeling, and to avoid debates about EBP, we focused more on participants’ actions and behaviour related to EBP and less on their beliefs; thinking that their behaviour would reflect attitudes and beliefs.

In summary, we do not claim that the descriptions in this study are the only possible interpretation of the data, but we believe that they reflect a common representation of students’ use of EBP during clinical education. Thus, the findings, and our suggestions contribute to important knowledge of some essential issues that needs to be taken into consideration when implementing EBP in future clinical physiotherapy education.

## Conclusions

Undergraduate health care students are expected to be competent evidence-based practitioners when they graduate. We found that third year physiotherapy students in clinical placements attempted to apply EBP, but struggled. Students were novices in clinical practice and tended to need basic and background information rather than research evidence. Participants seemed to believe that getting used to the new situation during clinical placements was more than enough for students. Consequently, practice experience and providing evidence-based patient management were perceived as incompatible approaches. Students perceived a lack of an EBP culture, and expressed a need for EBP role models during clinical placements. Our findings demonstrated that CIs are in a unique position to fulfil this role, and therefore, the clinical and the academic environment need to collaborate to ensure that CIs become competent in EBP. CIs role modelling EBP could be the right step towards clinically integrated teaching of EBP; in line with the existing evidence base. Future research need to investigate whether increasing EBP competency among CIs can enable CIs to role model EBP and to facilitate evidence-based patient management among health care undergraduate students.

## Competing interests

The authors declare that they have no competing interests.

## Authors’ contributions

NRO, PB and MWN designed the study. NRO collected the data. NRO, PB and KL performed the data analysis. NRO drafted the manuscript, and PB, KL and MWN contributed to this. All authors read and approved the final manuscript.

## Pre-publication history

The pre-publication history for this paper can be accessed here:

http://www.biomedcentral.com/1472-6920/13/52/prepub

## References

[B1] Dawes M Summerskill W Glasziou P Cartabellotta A Martin J Hopayian K Porzsolt F Burls A Osborne J Sicily statement on evidence-based practice BMC Med Educ 2005 5 1 1 10.1186/1472-6920-5-1 15634359 PMC544887

[B2] Coomarasamy A Khan KS What is the evidence that postgraduate teaching in evidence based medicine changes anything? A systematic review BMJ 2004 329 7473 1017 10.1136/bmj.329.7473.1017 15514348 PMC524555

[B3] Glasziou P Burls A Gilbert R Evidence based medicine and the medical curriculum BMJ 2008 337 a1253 10.1136/bmj.a1253 18815165

[B4] Dorsch JL Aiyer MK Meyer LE Impact of an evidence-based medicine curriculum on medical students’ attitudes and skills Journal of the Medical Library Association: JMLA 2004 92 4 397 406 15494754 PMC521510

[B5] Cayley WE Jr Evidence-based medicine for medical students: introducing EBM in a primary care rotation WMJ: official publication of the State Medical Society of Wisconsin 2005 104 3 34 37 15966630

[B6] Schilling K Wiecha J Polineni D Khalil S An interactive web-based curriculum on evidence-based medicine: design and effectiveness Fam Med 2006 38 2 126 132 16450235

[B7] Lai NM Nalliah S Information-seeking practices of senior medical students: the impact of an evidence-based medicine training programme Educ Heal 2010 23 1 151 20589599

[B8] Ilic D Tepper K Misso M Teaching evidence-based medicine literature searching skills to medical students during the clinical years: a randomized controlled trial Journal of the Medical Library Association: JMLA 2012 100 3 190 196 10.3163/1536-5050.100.3.009 22879808 PMC3411272

[B9] Lai NM Ramesh JC The product of outcome-based undergraduate medical education: competencies and readiness for internship Singapore Med J 2006 47 12 1053 1062 17139402

[B10] Pruskil S Burgwinkel P Georg W Keil T Kiessling C Medical students’ attitudes towards science and involvement in research activities: a comparative study with students from a reformed and a traditional curriculum Medical teacher 2009 31 6 e254 259 10.1080/01421590802637925 19811157

[B11] Cooper AL Elnicki DM Resource utilisation patterns of third-year medical students Clin Teach 2011 8 1 43 47 10.1111/j.1743-498X.2010.00393.x 21324072

[B12] Kim SC Brown CE Fields W Stichler JF Evidence-based practice-focused interactive teaching strategy: a controlled study J Adv Nurs 2009 65 6 1218 1227 10.1111/j.1365-2648.2009.04975.x 19445064

[B13] Morris J Maynard V Pilot study to test the use of a mobile device in the clinical setting to access evidence-based practice resources Worldviews on evidence-based nursing / Sigma Theta Tau International, Honor Society of Nursing 2010 7 4 205 213 19804588 10.1111/j.1741-6787.2009.00171.x

[B14] Zhang Q Zeng T Chen Y Li X Assisting undergraduate nursing students to learn evidence-based practice through self-directed learning and workshop strategies during clinical practicum Nurse Educ Today 2012 32 5 570 575 10.1016/j.nedt.2011.05.018 21664015

[B15] Long BK McEvoy MP Lewis LK Williams MT Olds TS Entry-Level Evidenced-Based Training in Physiotherapy Students: Does it Change Knowledge, Attitudes, and Behaviours? A longitudinal Study The Internet J Allied Health Sci Practice 2011 9 3

[B16] Manspeaker SA Van Lunen BL Turocy PS Pribesh S Hankemeier D Student Knowledge, Attitudes, and Use of Evidence-Based Concepts Following an Educational Intervention Athl Train Educ J 2011 6 2 88 98

[B17] Stronge M Cahill M Self-reported knowledge, attitudes and behaviour towards evidence-based practice of occupational therapy students in Ireland Occup Ther Int 2012 19 1 7 16 10.1002/oti.328 22183972

[B18] Florin J Ehrenberg A Wallin L Gustavsson P Educational support for research utilization and capability beliefs regarding evidence-based practice skills: a national survey of senior nursing students J Adv Nurs 2011 68 4 888 897 21950615 10.1111/j.1365-2648.2011.05792.x

[B19] Jonsen E Melender HL Hilli Y Finnish and Swedish nursing students’ experiences of their first clinical practice placement - A qualitative study Nurse Educ Today 2013 33 3 297 302 10.1016/j.nedt.2012.06.012 22795745

[B20] Bradley P Oterholt C Nordheim L Bjorndal A Medical students’ and tutors’ experiences of directed and self-directed learning programs in evidence-based medicine: a qualitative evaluation accompanying a randomized controlled trial Eval Rev 2005 29 2 149 177 10.1177/0193841X04269085 15731510

[B21] Ilic D Forbes K Undergraduate medical student perceptions and use of Evidence Based Medicine: a qualitative study BMC Med Educ 2010 10 58 10.1186/1472-6920-10-58 20718992 PMC2931522

[B22] Skoien AK Vagstol U Raaheim A Learning physiotherapy in clinical practice: student interaction in a professional context Physiother Theory Pract 2009 25 4 268 278 10.1080/09593980902782298 19418364

[B23] Utdannings- og forskningsdepartementet Rammeplan for fysioterapiutdanningen. [National Physiotherapy Curriculum] 2005 Oslo, Norway: Utdannings- og forskningsdepartementet. [The Ministry of Education and Research]

[B24] Thorne S Kirkham SR O’Flynn-Magee K The analytic challenge in interpretive description International Journal of Qualitative Methods 2004 3 2 1 11

[B25] Thorne SE Interpretive description 2008 Walnut Creek: Left Coast Press

[B26] Krueger RA Casey MA Focus groups: a practical guide for applied research 2000 Thousand Oaks, Calif: Sage

[B27] Lomborg K Ankersen L Fortolkende beskrivelse Klin Sygepleje 2010 24 1 7 15

[B28] Thorne S Kirkham SR MacDonald-Emes J Interpretive description: a noncategorical qualitative alternative for developing nursing knowledge Res Nurs Health 1997 20 2 169 177 10.1002/(SICI)1098-240X(199704)20:2<169::AID-NUR9>3.0.CO;2-I 9100747

[B29] King N Symon G, Cassell C Using Templates in the Thematic Analysis of Text Qualitative methods and analysis in organizational research: a practical guide 1998 London: Sage 256 270

[B30] Crabtree BF Miller WL Miller WL, Crabtree BF Using Codes and code manuals: A template organizing style of interpretation Doing qualitative research 1999 2 Thousand Oaks, Calif: Sage 163 177

[B31] King N Carroll C Newton P Dornan T ‘You can’t cure it so you have to endure it”: the experience of adaptation to diabetic renal disease Qual Health Res 2002 12 3 329 346 10.1177/104973202129119928 11918099

[B32] Brown CE Kim SC Stichler JF Fields W Predictors of knowledge, attitudes, use and future use of evidence-based practice among baccalaureate nursing students at two universities Nurse Educ Today 2010 30 6 521 527 10.1016/j.nedt.2009.10.021 19948369

[B33] Caldwell K Coleman K Copp G Bell L Ghazi F Preparing for professional practice: How well does professional training equip health and social care practitioners to engage in evidence-based practice? Nurse Educ Today 2007 27 6 518 528 10.1016/j.nedt.2006.08.014 17064821

[B34] Kamwendo K Tornquist K Do occupational therapy and physiotherapy students care about research? A survey of perceptions and attitudes to research Scand J Caring Sci 2001 15 4 295 302 10.1046/j.1471-6712.2001.00041.x 12453170

[B35] Waters D Crisp J Rychetnik L Barratt A The Australian experience of nurses’ preparedness for evidence-based practice J Nurs Manag 2009 17 4 510 518 10.1111/j.1365-2834.2009.00997.x 19531151

[B36] Shepard KF Hack LM Gwyer J Jensen GM Describing Expert Practice in Physical Therapy Qual Health Res 1999 9 6 746 758 10.1177/104973299129122252 10662257

[B37] Thomas A Saroyan A Dauphinee WD Evidence-based practice: a review of theoretical assumptions and effectiveness of teaching and assessment interventions in health professions Adv Health Sci Educ Theory Pract 2011 16 2 253 276 10.1007/s10459-010-9251-6 20922477

[B38] Copley J Allen S Using all the available evidence: perceptions of paediatric occupational therapists about how to increase evidence-based practice Int J Evid Based Healthc 2009 7 3 193 200 10.1111/j.1744-1609.2009.00137.x 21631861

[B39] Hannes K Filip S Jo G Bert A Obstacles to the implementation of evidence-based physiotherapy in practice: a focus group-based study in Belgium (Flanders) Physiother Theory Pract 2009 25 7 476 488 19925170 10.3109/09593980802661949

[B40] Straus SE Sackett DL Evidence-based medicine: how to practice and teach EBM 2005 3 Edinburgh: Elsevier Churchill Livingstone

[B41] Rappolt S Tassone M How rehabilitation therapists gather, evaluate, and implement new knowledge J Contin Educ Health Prof 2002 22 3 170 180 10.1002/chp.1340220306 12227239

[B42] Estabrooks CA Scott-Findlay S Winther C Lemieux-Charles L, Champagne F A Nursing and Allied Health Sciences Perspective on Knowledge Utilization Using Knowledge and Evidence in Health Care 2004 Toronto: University of Toronto Press 242 280

[B43] Bridges PH Bierema LL Valentine T The propensity to adopt evidence-based practice among physical therapists BMC Health Serv Res 2007 7 103 10.1186/1472-6963-7-103 17615076 PMC1929067

[B44] Grimmer-Somers K Lekkas P Nyland L Young A Kumar S Perspectives on research evidence and clinical practice: a survey of Australian physiotherapists Physiother Res Int 2007 12 3 147 161 10.1002/pri.363 17624895

[B45] Hannes K Leys M Vermeire E Aertgeerts B Buntinx F Depoorter AM Implementing evidence-based medicine in general practice: a focus group based study BMC Fam Pract 2005 6 37 10.1186/1471-2296-6-37 16153300 PMC1253510

[B46] Hannes K Norre D Goedhuys J Naert I Aertgeerts B Obstacles to implementing evidence-based dentistry: a focus group-based study J Dent Educ 2008 72 6 736 744 18519604

[B47] Hannes K Pieters G Goedhuys J Aertgeerts B Exploring barriers to the implementation of evidence-based practice in psychiatry to inform health policy: a focus group based study Community Ment Health J 2010 46 5 423 432 10.1007/s10597-009-9260-1 19888653

[B48] Hannes K Vandersmissen J De Blaeser L Peeters G Goedhuys J Aertgeerts B Barriers to evidence-based nursing: a focus group study J Adv Nurs 2007 60 2 162 171 10.1111/j.1365-2648.2007.04389.x 17877563

[B49] Iles R Davidson M Evidence based practice: a survey of physiotherapists’ current practice Physiother Res Int 2006 11 2 93 103 10.1002/pri.328 16808090

[B50] Jette DU Bacon K Batty C Carlson M Ferland A Hemingway RD Hill JC Ogilvie L Volk D Evidence-based practice: beliefs, attitudes, knowledge, and behaviors of physical therapists Phys Ther 2003 83 9 786 805 12940766

[B51] Kamwendo K What do Swedish physiotherapists feel about research? A survey of perceptions, attitudes, intentions and engagement Physiother Res Int 2002 7 1 23 34 10.1002/pri.238 11992982

[B52] Metcalfe C Lewin E Wisher S Perry S Bannigan K Mofett JK Barriers to Implementing the Evidence Base in Four NHS Therapies. Dietitians, occupational therapists, speech and language therapist Physiotherapy 2001 87 8 433 441 10.1016/S0031-9406(05)65462-4

[B53] Palfreyman S Tod A Doyle J Comparing evidence-based practice of nurses and physiotherapists Br J Nurs 2003 12 4 246 253 12671571 10.12968/bjon.2003.12.4.11165

[B54] Upton D Upton P Knowledge and use of evidence-based practice by allied health and health science professionals in the United Kingdom J Allied Health 2006 35 3 127 133 17036666

[B55] Thompson DS O’Leary K Jensen E Scott-Findlay S O’Brien-Pallas L Estabrooks CA The relationship between busyness and research utilization: it is about time J Clin Nurs 2008 17 4 539 548 10.1111/j.1365-2702.2007.01981.x 18205684

[B56] Haynes B Of studies, syntheses, synopses, summaries, and systems: the “5S” evolution of information services for evidence-based healthcare decisions In 2007 10 6 7 10.1136/ebn.10.1.6 17218282

[B57] Maher CG Moseley AM Sherrington C Elkins MR Herbert RD A description of the trials, reviews, and practice guidelines indexed in the PEDro database Phys Ther 2008 88 9 1068 1077 10.2522/ptj.20080002 18635670

[B58] Salbach NM Jaglal SB Korner-Bitensky N Rappolt S Davis D Practitioner and organizational barriers to evidence-based practice of physical therapists for people with stroke Phys Ther 2007 87 10 1284 1303 discussion 1304–1286 10.2522/ptj.20070040 17684088

[B59] Barnard S Wiles R Evidence-based Physiotherapy: Physiotherapists’ attitudes and experiences in the Wessex area Physiotherapy 2001 87 3 115 124 10.1016/S0031-9406(05)61078-4

[B60] Roskell CHA Wildman S The theory-practice gap and physiotherapy in the UK: Insight from the nursing experience Physiother Theory Pract 1998 14 223 233 10.3109/09593989809057168

[B61] Stube JE Jedlicka JS The acquisition and integration of evidence-based practice concepts by occupational therapy students Am J Occup Ther 2007 61 1 53 61 10.5014/ajot.61.1.53 17302105

[B62] Manspeaker SA Van Lunen B Implementation of Evidence-Based Practice Concepts in Undergraduate Athletic Training Education: Experiences of Select Educators Athl Train Educ J 2010 5 2 51 60 10.4085/1062-6050-46.5.514 PMC3418958 22488139

[B63] Bleakley A Bligh J Browne J Bleakley A, Bligh J, Browne J Socio-Cultural Learning Theories Medical Education for the Future Identity, Power and Location 2011 Dordrecht, Netherlands: Springer 43 60

[B64] Ohman A Solomon P Finch E Career Choice and Professional Preferences in a Group of Canadian Physiotherapy Students Adv Physiother 2002 4 16 22 10.1080/140381902317303177

[B65] Sabus C The Effects of Modeling Evidence-Based Practice During the Clinical Internship J Phys Ther Educ 2008 22 3 74 84

[B66] Laitinen-Väänänen S The construction of supervision and physiotherapy expertise. A qualitative study of physiotherapy students learning sessions in clinical education Doctoral thesis 2008 Jyväskylä, Finland: University of Jyväskylä

[B67] Laitinen-Vaananen S Talvitie U Luukka MR Clinical supervision as an interaction between the clinical educator and the student Physiother Theory Pract 2007 23 2 95 103 10.1080/09593980701212018 17530539

[B68] Helse- og omsorgsdepartementet Oppdragsdokument 2011 Helse Vest RHF [The assignment document 2011 for Western Norway Regional Health Authority] 2011 Oslo, Norway: Helse- og omsorgsdepartementet [The Ministry of Health and Care Services]

[B69] Høgskolen i Bergen Strategisk plan for Høgskolen i Bergen [The Strategic Plan of Bergen University College] Bergen, Norway: HiB [Bergen University College]. [Internet] http://www.hib.no/om/sentrale-dokumenter/strategi-og-planer/strategisk_plan/index.htm

